# Characterization of TIM-3 Expression and Its Correlation with TNF-*α* and IFN-*γ* in Patients with Surgically Resected Lung Adenocarcinoma

**DOI:** 10.1155/2023/2352945

**Published:** 2023-02-21

**Authors:** Jian Tan, Piao Shen, Xingping Yang, Weijie Cai, Hongying Liao

**Affiliations:** ^1^Department of Thoracic Surgery, Thoracic Cancer Center, The Sixth Affiliated Hospital of Sun Yat-sen University, Guangzhou, Guangdong, China 510665; ^2^Department of Thoracic Surgery, Guangzhou Medical University Affiliated Cancer Hospital, Guangzhou, Guangdong, China 510095

## Abstract

**Objective:**

T cell immunoglobulin and mucin-containing protein-3 (TIM-3) is an important immune checkpoint, but its role in lung cancer is still not clear. In this study, we investigated TIM-3 protein expression and its correlation with TNF-*α* and IFN-*γ* by examining the tissues of patients with lung adenocarcinoma.

**Methods:**

We detected the mRNA quantity of TIM-3, TNF-*α*, and IFN-*γ* in 40 surgically resected specimens from patients with lung adenocarcinoma by real-time quantitative polymerase chain reaction (qRT-PCR). The protein expression of TIM-3, TNF-*α*, and IFN-*γ* was assessed in normal tissues, paracarcinoma tissues, and tumor tissues by western blotting, respectively. The relevance between the expression and clinicopathological information of the patients was analyzed.

**Results:**

The results showed that the expression level of TIM-3 was higher in tumor tissues than normal tissues and paracancerous tissues (*P* < 0.05). On the contrary, the expression of TNF-*α* and IFN-*γ* in tumor tissues was lower than normal tissues and paracarcinoma tissues (*P* < 0.05). However, the expression levels of IFN-*γ* mRNA were not observed to be significantly different between cancerous tissues and adjacent tissues. While TIM-3 protein expression in cancer tissues of patients with lymph node metastasis was higher than in patients without metastasis, the expression of TNF-*α* and IFN-*γ* was lower (*P* < 0.05). Importantly, the expression of TIM-3 was negatively correlated with the expression of TNF-*α* and IFN-*γ*, and the expression of TNF-*α* was found to be positively correlated with IFN-*γ* in the patient.

**Conclusion:**

The high expression of TIM-3, the low expression of TNF-*α* and IFN-*γ*, and the synergistic effect of TNF-*α* and IFN-*γ* in patients with lung adenocarcinoma were closely related to poor clinicopathological characteristics. Overexpression of TIM-3 may play an important role in the relationship between TNF-*α* and IFN-*γ* secretion and poor clinicopathological characteristics.

## 1. Introduction

Lung cancer is the most frequent diagnosed cancer and remains a leading cause of cancer death in the world [1, 2]. Lung adenocarcinomas (LUAD), the commonest histological subtype of lung cancers, varies from 50% to 60% of lung cancer, of which a total of 5-year survival reaches approximately 19% [3, 4]. The majority of patients with lung cancers are detected at its advanced stage, indicating that the most of patients sustain unfounded metastases at this period of the cancers [5]. As the common metastatic pathway of LUAD, lymph node metastasis is an important factor in predicting and determining the prognosis of the patients [6].

The extended life of non-small-cell lung cancer (NSCLC) patients has been achieved today by the introduction of molecular therapy in those who are positive for EGFR mutations because they receive TKI inhibitors [7]. Research is still needed and that immunology plays a big role in cancer, especially cytokine research, and the application of immunomodulatory therapy [8]. Today, various attempts at immunotherapy are applied, of which only one is examined here. Therefore, the large number of research currently in applying immunotherapeutic agents is a testament to the tremendous advances they are making in cancer remedy, especially the prognosis of the advanced sickness. However, the clinical outcomes of a large part of patients still are unsatisfactory. Exploring new and efficient immune checkpoints is extremely urgent [9].

T cell immunoglobulin domain and mucin domain 3 (TIM-3) is a member of TIM family proteins, that is, type I cell surface glycoproteins, and it is reported repeatedly that the protein has relation to autoimmune reactions [10, 11]. A firm connection between upregulated TIM-3 expression and exhausted immune response was initially established in multiple chronic inflammation [12]. The origin of malignancy may lie in chronic infection, and that infection is the key aspect of cancer progression [13]. TIM-3 recently has showed as a potential immune checkpoint target in the therapy of cancer, but much of the detail regarding its specific mechanism remains unclear.

Immune system has been testified to be an effective implement capable of inhibiting the merisis and metastases of cancer. Inflammation is a necessary and vital procedure of the immune system for combatting against tumors. Tumor necrosis factor-*α* (TNF-*α*) and interferon-*γ* (IFN-*γ*) are important inflammations that are related to all stage of different types of tumors [14–16]. However, the roles of the TNF-*α* and IFN-*γ* expressions in regulating merisis and metastases of cancer in LUAD remain to be determined.

Therefore, the present study is aimed at investigating the clinicopathological features of TIM-3, TNF-*α*, and IFN-*γ* expressions and the correlation between their expressions in patients with surgically resected LUAD.

## 2. Material and Methods

### 2.1. Patients and Clinicopathologic Variables

In the present study, a total of 40 consecutive patients with LUAD were prospectively collected from September 2018 to June 2020 in the local Affiliated Cancer Hospital and Institute of Guangzhou Medical University, China. There were 22 males and 18 females with a mean age of 59.67 ± 1.42 years, ranging from 39 to 78 years old. The samples were collected from each participants during the same time, including the LUAD normal lung tissues (>5.0 cm beyond the cancer tissue), paracancerous tissues (<2.0 cm beyond the cancer tissue), and radical cancerous tissues. None of the patients received a previous history of preoperative chemotherapy and radiotherapy. This investigation was approved by the ethics committee of the Affiliated Cancer Hospital and Institute of Guangzhou Medical University. The informed written consent was obtained from each patient. Ethical approval is No. 118 (2015).

### 2.2. qRT-PCR

RNA extraction from tissue sample was implemented using the TRIzol reagents (Takara, Japan) according to the manufacture protocol. The cDNA of mRNA was reverse-transcribed utilizing PrimeScript RT Master Mix (Takara, Japan). TB Green Premix Ex Taq II (Takara, Japan) was used to carry out qRT-PCR on the LightCycler 96 system (Roche, Switzerland). In addition, PCR thermal cycle parameters were as follows: firstly incubated at 95°C for 2 min, followed by 40 cycles of 95°C for 5 s and 60°C for 30 s. According to the sequence of human TIM-3, TNF-*α*, IFN-*γ*, and *β*-actin in NCBI GenBank, Premier 6.0 software (Premier, USA) was utilized for the design of the primer. Their relative mRNA expression levels were calculated by 2^−*ΔΔ*CT^ and normalized to *β*-actin expression in each sample.

### 2.3. Western Blot Analysis

The normal tissues and adjacent paracancerous and cancerous tissues were lysed with ice-cold RIPA buffer containing protease inhibitor, in accordance with the manufacturer's protocol applying the bicinchoninic acid assay (BCA) (Thermo Scientific, USA) methods for total protein, using BSA as standards. Meanwhile, the proteins were denatured at 100°C for 5 min. A total of 30 ng of proteins was separated in 12.5% SDS-PAGE and transferred to a PVDF membrane. Then, it was blocked with 5% skim milk for 2 h. The following antibodies were used in the study: rabbit anti-TIM-3 antibody (Abcam, USA), anti-TNF-*α* antibody (Abcam, USA), anti-IFN-*γ* antibody (Abcam, USA), and mouse anti-*β*-actin antibody (Proteintech, China). The PVDF membranes were incubated with TIM-3, TNF-*α*, IFN-*γ*, and *β*-actin primary antibodies at 4°C overnight. The secondary antibodies (CST, USA) were used to incubate with the membranes for 1 h at room temperature. After washing with TBST, the membranes were visualized in ECL developing solution. Band intensity was scanned by ImageJ software. Quantification was determined by estimating the intensity value ratios of the reference *β*-actin and the target gene.

### 2.4. Statistical Analysis

SPSS version 26.0 statistical software (SPSS, USA) was used to perform all data. The data were expressed as mean ± standard deviation (SD), respectively, and the corresponding comparison tests were the *t*-test, Wilcoxon signed rank test, or Mann-Whitney *U* test. Spearman's rank correlation coefficients (Rs) were used to analyze the correlations between TIM-3, TNF-*α*, and IFN-*γ* expressions. *P* values < 0.05 (two-side) were considered statistically significant. Graphs were made utilizing GraphPad Prism 8.0 (GraphPad Prism, USA).

## 3. Results

### 3.1. Baseline Characteristic of the Included Subjects

In this study, there were 55% males and 45% females in the case group with a mean age of 59.67 ± 1.42 years. In addition, the study group included four patients with pleural metastasis by postoperative pathology. Most of patients (52.5%) had right-sided tumors. In the case group, tumor size was T1 (37.5%) and T2 to T4 (62.5%). Regarding TNM stages, 47.5% patients were in stage I and 52.5% in stage II to IV. Of all, 57.5% were nonsmokers and BMI ≤ 23.9, 62.5% were no lymphatic metastasis, and 60% were CEA > 5. The patient and tumor characteristic are presented in Table 1.

### 3.2. TIM-3, TNF-*α*, and IFN-*γ* Protein Expression Levels in LUAD Tissue

**Figure 1:**
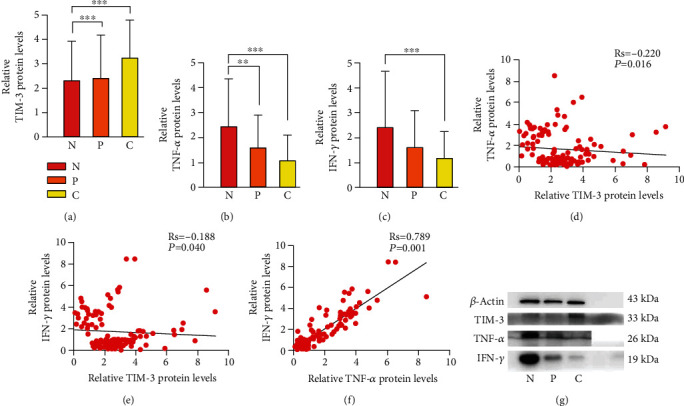
Expression of TIM-3, TNF-*α*, and IFN-*γ* protein in LUAD. (a) High TIM-3 protein expression in cancer tissue (C, *n* = 40) of LUAD patients compared with paracancerous tissue (P, *n* = 40) and normal controls (N, *n* = 40). (b) TNF-*α* protein expression was significantly lowered in LUAD tissues (*n* = 40) compared with paracancerous tissues (*n* = 40) and normal tissues (*n* = 40). (c) IFN-*γ* protein expression was significantly lowered in LUAD tissues (*n* = 40) compared with normal tissues (*n* = 40). (d–f) Scatter plot of TIM-3, TNF-*α*, and IFN-*γ* protein expressions in LUAD (*n* = 40). (g) Western blot analysis expression of TIM-3, TNF-*α*, *β*-actin, and IFN-*γ* in LUAD. ^∗^*P* < 0.05, ^∗∗^*P* < 0.01, and ^∗∗∗^*P* < 0.001.

Specimens were assessed by western blot. There was a positive raise trend of TIM-3 protein expression in cancer tissue of LUAD patients compared with paracancerous tissue and normal controls (*P* < 0.05) (Figures 1(a) and 1(g)). Meanwhile, TNF-*α* protein expression was significantly lowered in LUAD tissues compared with paracancerous tissues and normal tissues (*P* < 0.05) (Figures 1(b) and 1(g)). Lower protein expression of IFN-*γ* was detected in the cancer tissues than normal tissue (*P* < 0.05) (Figures 1(c) and 1(g)).

### 3.3. Relationship between TIM-3, TNF-*α*, or IFN-*γ* Protein Expression and the Clinicopathologic Parameters of LUAD

To verify the relationship of TIM-3, TNF-*α*, or IFN-*γ* protein expression with the clinicopathologic parameters of LAUD, gender, age, TNM stages, lymph node metastasis, smoke, CEA, BMI, location, tumor size, and date of operation were investigated in this study (Tables 1–3). Although the expression of TIM-3 in cancer tissue was enhanced as mentioned above, the overexpression was found in cancer tissue from patients significantly (*P* < 0.05). No significant difference was found in paracancerous tissues. Meanwhile, TNF-*α* and IFN-*γ* protein expressions in the cancer tissues were significantly low in patients with lymph node metastasis (*P* < 0.05). The same result in paracancerous sample was found but did not reach statistical significance. For the other parameters, no apparent statistical differences were found.

### 3.4. Correlations between TIM-3, TNF-*α*, or IFN-*γ* Protein in LUAD Expression

**Figure 2:**
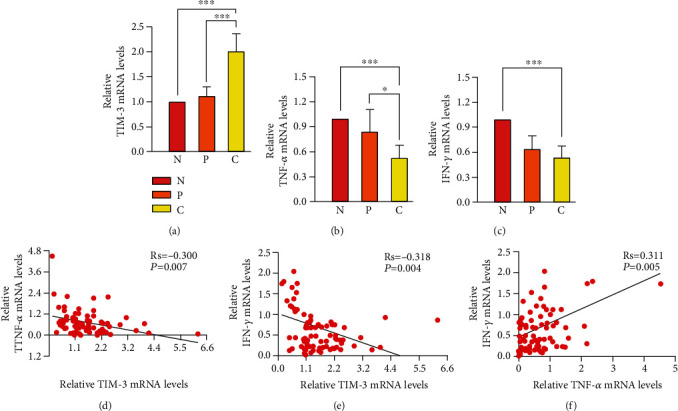
Expression of TIM-3, TNF-*α*, and IFN-*γ* mRNA and their correlation in LUAD. (a) High TIM-3 mRNA expression in cancer tissue (*n* = 40) of LUAD patients compared with paracancerous tissue (*n* = 40) and normal controls (*n* = 40). (b) TNF-*α* mRNA expression was significantly lowered in LUAD tissues (*n* = 40) compared with paracancerous tissues (*n* = 40) and normal tissues (*n* = 40). (c) IFN-*γ* mRNA expression was significantly lowered in LUAD tissues (*n* = 40) compared with normal tissues (*n* = 40). (d–f) Scatter plot of TIM-3, TNF-*α*, and IFN-*γ* mRNA expressions in LUAD (*n* = 40). ^∗^*P* < 0.05, ^∗∗^*P* < 0.01, and ^∗∗∗^*P* < 0.001.

The study showed that overexpression of TIM-3, lower TNF-*α*, and IFN-*γ* protein expressions in cancer tissue was relative to lymph node metastasis. Furthermore, to observe the correlation between the 3 characteristics, we analyze the relative mRNA expression of TIM-3, TNF-*α*, and IFN-*γ* from the tissues of patients by the relative quantification method and correlations between their expressions in LUAD. Although TIM-3, TNF-*α*, and IFN-*γ* mRNA levels were relative to lymph nodes metastasis, there were no statistical differences in the cancer tissue and paracancerous tissue (*P* > 0.05). The mRNA expression of the genes was consistent with protein expression. The corresponding result revealed that the enhanced levels of TIM-3 mRNA in cancer tissues were found compared with normal tissues and paracancerous tissues (*P* < 0.05) (Figure 2(a)). TNF-*α* showed a low expression in cancer tissues than normal tissues and paracancerous tissues (*P* < 0.05) (Figure 2(b)). A similar result was also detected in the comparison of IFN-*γ* mRNA levels between the cancer tissues and normal tissues (*P* < 0.05) (Figure 2(c)). Gene expression levels are associated with expression where its levels were equal to 1 in normal tissue. Moreover, the protein and mRNA expressions of TIM-3 were significantly negatively correlated with expression of TNF-*α* in LUAD (Rs = −0.220, *P* = 0.016, protein) (Rs = −0.300, *P* = 0.007, mRNA) (Figures 1(d) and 2(d)). The same result was also found between the expressions of TIM-3 and IFN-*γ* in LUAD (Rs = −0.188, *P* = 0.040, protein) (Rs = −0.318, *P* = 0.004, mRNA) (Figures 1(e) and 2(e)). Then, the protein and mRNA levels of TNF-*α* were significantly positively associated with levels of IFN-*γ* in LUAD (Rs = 0.789, *P* = 0.001, protein) (Rs = 0.311, *P* = 0.005, mRNA) (Figures 1(f) and 2(f)).

## 4. Conclusions

In this study, we detected the protein expression of TIM-3 in normal, paracancerous, and tumor tissues of lung adenocarcinoma. We found that TIM-3 expression was higher in carcinoma tissues than in paracancerous tissues and normal tissues. Importantly, TIM-3 expression was higher in carcinoma tissues of patients with lymph node metastasis than in patients without lymph node metastasis, and the same results were obtained in paracancerous tissues but were not statistically significant. There were reports that upregulation of TIM-3 expression is closely associated with prognosis in patients with bone cancer [17]. Our study showed that high expression of Tim-3 was closely related to lymph node metastasis.

To further analyze the relationship between the high expression of Tim-3 and lymph node metastasis, we detected the expression of TNF-*α* and IFN-*γ* proteins in normal tissues, paracancerous tissues, and tumor tissues in patients with lung adenocarcinoma. We found that the expressions of TNF-*α* and IFN-*γ* in cancer tissues were lower than those in paracancerous tissues and normal tissues. Interestingly, the expressions of TNF-*α* and IFN-*γ* in cancer tissues of patients with no lymph node metastasis were higher than those in patients with lymph node metastasis. Studies have shown that when there is a high concentration of TNF-*α* in tumors, TNF-*α* may selectively destroy blood vessels in tumor tissues and promote T cell immune response, but low concentrations of TNF-*α* may promote tumor progression and lead to poor prognosis [18]. If the function of peripheral blood cells to produce cytokines among them IFN-*γ* is examined, then the capacity of the cells to produce cytokines is reduced. This indicates damage to the function of cells of the immune system, and the median survival of patients with low IFN-*γ* expression is significantly lower than that of patients with high IFN-*γ* expression [19, 20]. Our study showed that low expression of TNF-*α* and IFN-*γ* was closely associated with lymph node metastasis.

Subsequently, we detected the mRNA expression of Tim-3, TNF-*α*, and IFN-*γ* in normal, paracancerous, and tumor tissues of patients with lung adenocarcinoma and analyzed the correlation between the expression of Tim-3 and TNF-*α* and IFN-*γ* in lung adenocarcinoma. The results showed that Tim-3 was negatively correlated with the protein and mRNA expressions of TNF-*α* and IFN-*γ*, respectively. In other word, in patients with lung adenocarcinoma, the expression of TNF-*α* and IFN-*γ* decreased with the upregulation of Tim-3 expression. The results showed that TIM-3 acted on the polarization of M2 macrophages of STAT1 (signal transcription and activator of transcription 1) to promote the antitumor effect of macrophages in malignant tumors. In addition, blocking STAT1 inhibits the secretion of TNF-*α* and IFN-*γ* by immune cells, leading to tumor growth and metastasis [14, 21, 22]. This study suggested that high expression of Tim-3 may inhibit the secretion of TNF-*α* and IFN-*γ* by immune cells, leading to tumor progression.

In addition, the results showed that the mRNA expressions of TNF-*α* and IFN-*γ* in cancer tissues were lower than those in paracancerous tissues and normal tissues. We further analyzed the correlation between the two inflammatory factors, and our data showed that the protein and mRNA of the two inflammatory factors were positively correlated, indicating that the expressions of TNF-*α* and IFN-*γ* in lung adenocarcinoma were synergistic. It has been reported that a small amount of TNF-*α* produced by macrophages will stimulate NK cells to secrete a small amount of IFN-*γ*, and the IFN-*γ* produced by NK cells will react on the macrophages to produce TNF-*α*. This form of mutual stimulation forms a benign proliferation cycle, which is conducive to the immune system in the antitumor process; when the adaptive immunity has not been activated, the innate immunity can have the effective killing ability on the tumor [23, 24]. This study showed that TNF-*α* and IFN-*γ* synergistically promoted lymph node metastasis.

Finally, there is still a question worth pondering. Our data suggest that the expression of Tim-3 mRNA in lung adenocarcinoma tissues is higher than that in paracancerous tissues and normal tissues, but when the expression of Tim-3, TNF-*α*, and IFN-*γ* mRNA is analyzed separately with lymph node metastasis, the results show no statistical significance. Studies have reported that TIM-3 regulates tumors by STAT1, and the transcriptional function of STAT1 selectively regulates mRNA translation to regulate tumor proliferative and prosurvival properties [25]. Whether the high expression of Tim-3 in lung adenocarcinoma can inhibit the expression of TNF-*α* and IFN-*γ* through signal transduction or regulation of translation remains to be further explored. Our study showed that the high expression of Tim-3, low expression of TNF-*α* and IFN-*γ*, and the synergistic effect of TNF-*α* and IFN-*γ* in patients with lung adenocarcinoma were closely related to poor clinicopathological characteristics. Overexpression of Tim-3 may play an important role in the relationship between TNF-*α* and IFN-*γ* secretion and worse clinicopathological parameters such as lymph node metastasis. Anyway, we can learn more from the expression characteristics of Tim-3, TNF-*α*, and IFN-*γ* that the potential mechanism of them in the immune system against tumor.

## Figures and Tables

**Table 1:** Correlation between expression of TIM-3 protein and clinicopathologic parameters.

Variables	Number	Paracancerous $\left(\bar{x}\pm s\right)$	*P* value	Cancerous $\left(\bar{x}\pm s\right)$	*P* value
TNM stages					
I	19	2.289 ± 0.504	0.076	2.937 ± 0.367	0.219
II-IV	21	2.536 ± 0.286		3.543 ± 0.320	
Gender					
Female	18	2.882 ± 0.484	0.065	3.546 ± 0.297	0.287
Male	22	2.039 ± 0.305		3.017 ± 0.369	
Age					
≥60	24	2.200 ± 0.342	0.194	3.280 ± 0.367	0.566
<60	16	2.746 ± 0.477		3.217 ± 0.276	
Smoke					
Yes	17	2.034 ± 0.380	0.087	2.890 ± 0.468	0.238
No	23	2.703 ± 0.393		3.526 ± 0.239	
T stages					
T1	15	1.960 ± 0.243	0.426	3.268 ± 0.410	0.968
T2-T4	25	2.694 ± 0.417		3.247 ± 0.309	
Lymph node metastasis					
Yes	15	2.708 ± 0.362	0.067	3.975 ± 0.375	0.020
No	25	2.245 ± 0.392		2.823 ± 0.291	
Location					
Left	19	2.787 ± 0.543	0.882	3.016 ± 0.293	0.357
Right	21	2.085 ± 0.197		3.471 ± 0.381	
Date of operation					
<June	15	1.747 ± 0.279	0.049	3.183 ± 0.406	0.822
≥June	25	2.822 ± 0.397		3.299 ± 0.311	
CEA					
>5	16	2.608 ± 0.586	0.581	3.301 ± 0.426	0.818
≤5	24	2.292 ± 0.264		3.224 ± 0.298	
BMI					
>23.9	17	3.112 ± 0.565	0.116	3.512 ± 0.291	0.372
≤23.9	23	1.906 ± 0.203		3.065 ± 0.366	

**Table 2:** Correlation between expression of TNF-*α* protein and clinicopathologic parameters.

Variables	Number	Paracancerous $\left(\bar{x}\pm s\right)$	*P* value	Cancerous $\left(\bar{x}\pm s\right)$	*P* value
TNM stages					
I	19	1.722 ± 0.315	0.903	0.774 ± 0.119	0.171
II-IV	21	1.473 ± 0.280		1.356 ± 0.276	
Gender					
Female	18	1.487 ± 0.339	0.447	1.137 ± 0.253	0.463
Male	22	1.667 ± 0.262		1.032 ± 0.210	
Age					
≥60	24	1.686 ± 0.281	0.825	1.065 ± 0.235	0.440
<60	16	1.450 ± 0.311		1.101 ± 0.200	
Smoke					
Yes	17	1.700 ± 0.322	0.774	0.959 ± 0.198	0.516
No	23	1.512 ± 0.277		1.169 ± 0.240	
T stages					
T1	15	1.272 ± 0.273	0.224	1.088 ± 0.300	0.989
T2-T4	25	1.783 ± 0.286		1.074 ± 0.188	
Lymph node metastasis					
Yes	15	1.145 ± 0.221	0.476	0.725 ± 0.941	0.023
No	25	1.860 ± 0.295		1.671 ± 0.355	
Location					
Left	19	1.760 ± 0.315	0.409	1.128 ± 0.266	0.968
Right	21	1.440 ± 0.278		1.035 ± 0.195	
Date of operation					
<June	15	1.821 ± 0.369	0.769	1.047 ± 0.253	0.922
≥June	25	1.454 ± 0.250		1.099 ± 0.211	
CEA					
>5	16	1.962 ± 0.351	0.098	1.351 ± 0.354	0.782
≤5	24	1.345 ± 0.249		0.899 ± 0.122	
BMI					
>23.9	17	1.409 ± 0.284	0.712	1.222 ± 0.274	0.359
≤23.9	23	1.726 ± 0.296		0.974 ± 0.194	

**Table 3:** Correlation between expression of IFN-*γ* protein and clinicopathologic parameters.

Variables	Number	Paracancerous $\left(\bar{x}\pm s\right)$	*P* value	Cancerous $\left(\bar{x}\pm s\right)$	*P* value
TNM stages					
I	19	1.628 ± 0.299	0.924	1.401 ± 0.262	0.198
II-IV	21	1.622 ± 0.356		1.000 ± 0.207	
Gender					
Female	18	1.667 ± 0.382	0.828	1.233 ± 0.246	0.605
Male	22	1.591 ± 0.291		1.157 ± 0.231	
Age					
≥60	24	1.588 ± 0.287	0.761	1.330 ± 0.243	0.377
<60	16	1.681 ± 0.399		0.982 ± 0.198	
Smoke					
Yes	17	1.680 ± 0.360	0.859	1.265 ± 0.290	0.795
No	23	1.585 ± 0.310		1.136 ± 0.200	
T stages					
T1	15	1.240 ± 0.263	0.410	1.194 ± 0.245	0.727
T2-T4	25	1.856 ± 0.330		1.189 ± 0.226	
Lymph node metastasis					
Yes	15	1.334 ± 0.387	0.442	0.727 ± 0.131	0.043
No	25	1.800 ± 0.289		1.469 ± 0.239	
Location					
Left	19	1.675 ± 0.313	0.818	1.145 ± 0.258	0.394
Right	21	1.580 ± 0.346		1.233 ± 0.221	
Date of operation					
<June	15	1.921 ± 0.423	0.394	1.320 ± 0.290	0.494
≥June	25	1.447 ± 0.271		1.113 ± 0.205	
CEA					
>5	16	1.967 ± 0.379	0.167	1.283 ± 0.311	0.978
≤5	24	1.397 ± 0.289		1.129 ± 0.189	
BMI					
>23.9	17	1.334 ± 0.249	0.712	0.935 ± 0.126	0.774
≤23.9	23	1.840 ± 0.357		1.380 ± 0.270	

## Data Availability

The data used to support the findings of this study are included within the article.
